# Microscopic Factors Affecting the Performance of Pervious Concrete

**DOI:** 10.3390/ma17071479

**Published:** 2024-03-24

**Authors:** Qin Liu, Hu Li, Qianli Cao, Di Ke, Shiyang Yin, Qinpeng Li

**Affiliations:** 1 School of Architecture and Engineering, Chang’an University, Xi’an 710061, China; 2021128059@chd.edu.cn (H.L.); 2022128052@chd.edu.cn (Q.C.); m18225541896@163.com (D.K.); 2 School of Water Resources and Hydroelectric Engineering, North China Electric Power University, Beijing 102206, China; l19180861421@163.com; 3 Shandong Urban Rural Planning and Design Research Institute Co., Ltd., Jinan 250014, China; l13340735772@163.com

**Keywords:** pervious concrete, image analysis, fine analysis, permeability coefficient, compressive strength

## Abstract

The impacts of various aggregate particle sizes and cement contents on the internal structure of pervious concrete were investigated. Accordingly, test blocks with different aggregate particle sizes and cement contents were dissected and photographed. Subsequently, the captured images were processed using the ImageJ software (1.53i) to analyze the profiles of the test blocks and identify the internal mesoscopic parameters of the pervious concrete. This study discusses the relationship between microscopic parameters and macroscopic factors based on experimental results. It also fits functional equations linking the permeability coefficient with pore parameters, matrix parameters, and compressive strength. The results indicated that, as the aggregate size increased, the internal pore diameter of the pervious concrete increased, whereas the total area and width of the cement matrix decreased. This resulted in a low permeability coefficient and high compressive strength of the test block. Increasing the cement content in pervious concrete reduced the porosity and increased the width and area of the internal matrix. Consequently, the permeability coefficient decreased, and the compressive strength of the test block increased.

## 1. Introduction

The construction of sponge cities can effectively resolve two major problems: frequent urban flooding and water shortages. First, sponge cities enable the infiltration of road surface water, thereby reducing the pressure of flood discharge through the urban pipe network. Second, they can collect rainwater and replenish groundwater resources. As a green building material, pervious concrete is an important part of sponge city construction. It is currently widely used on non-main roads in sponge cities, and it can be seen everywhere on sidewalks and parking lots [1]. Pervious concrete has played an important role in alleviating the problem of urban waterlogging; at the same time, with the continuous accumulation of blockages, such as those caused by sediment, its permeability is reduced, and eventually, it evolves into impervious concrete pavement. As a result, its permeability is basically lost, and it has no application value in practical engineering. Therefore, it is of great significance to study the mechanical properties of pervious concrete for the realization of low-carbon urban development.

Pervious concrete, which is also known as porous concrete, usually has a porosity between 10% and 25%, a lower strength than that of ordinary concrete, and a permeability coefficient that is generally greater than 0.5 mm/s. Basic experimental research on pervious concrete is mainly performed by changing the aggregate particle size/gradation, ash-to-bone ratio, water–cement ratio, target porosity, and other factors to prepare it to meet the needs of different occasions. When the target porosity, aggregate size, cement content, and other raw material parameters are fixed, with the increase in the water–cement ratio, the strength of pervious concrete first rises and then decreases, and its porosity and permeability coefficient gradually decrease; when the water–cement ratio is optimal, the test results are roughly close, but there is still a certain fluctuation (0.28–0.33). The strength of pervious concrete is greatly affected by the properties of its raw materials and the preparation technology used, so there are certain differences. At present, the research on pervious concrete mainly involves the improvement of its properties by changing the aggregate particle size/gradation, water–cement ratio/ash–bone ratio, target porosity, and external materials. These include studies of the design of the mix ratio [2,3,4,5], the influence of the aggregate particle size [6,7,8,9,10] and type [11,12] on the performance and porosity [13,14,15,16], the relationship between permeability and the mechanical properties of pervious concrete [17,18], and fatigue performance [19,20,21,22]. However, most scholars have not extensively investigated the fine-scale parameters of pervious concrete to analyze its pore structure characteristics, except through electron microscopy [23], computed tomography scanning reconstruction [24], and the piezomercury method [25].

Most studies only focused on the effects of the pore structure characteristics, such as the planar porosity [26], pore diameter [24], and curvature [25], on the permeability coefficient and compressive strength of pervious concrete without considering the role of cement in the specimen matrix. In reality, the pore parameters considerably influence the permeability coefficient of pervious concrete, whereas the bonded matrix among aggregates determines its strength.

Based on this, laboratory testing and theoretical analysis were combined here to study the effects of the aggregate particle size and cement content on the pore structure and bonded matrix of pervious concrete, and its mesoscopic parameters were determined. Based on the experimental data, the relationship between the mesoscopic parameters and the basic properties of pervious concrete was established by fitting an equation. The results of this research provide a basis for the realization of low-carbon urban development.

## 2. Experimental Process

### 2.1. Specimen Preparation

#### 2.1.1. Test Materials

(1) Cement: 42.5R ordinary silicate cement produced by the BBMG Corporation (Beijing, China), as shown in Table 1.

(2) Coarse aggregate: Three types of single-size crushed stone with sizes in the ranges of 2.36–4.7, 4.75–9.5, and 9.5–16 mm, as shown in Table 2.

(3) Water: tap water.
(4) Fine sand: mechanism sand.
(5) Mineral powder.
(6) Polypropylene fiber, as shown in Table 3.

#### 2.1.2. Specimen Casting

Pervious concrete specimens were prepared according to the mix ratios listed in Table 4. The specimens were used to investigate the impacts of factors such as the particle size, proportions, and admixture on the performance of pervious concrete. Cement, water, fine sand, and polypropylene fiber were placed in a mixer and stirred at a constant speed for 1 min; then, the natural coarse aggregate was added to the mixer and stirred for 30 s, followed by the addition of the remaining raw materials, which were stirred for 1 min. After the mixing was completed, the concrete was poured into a mold and vibrated on a vibrating table for 15 s. The cement slurry floating on the surface of the specimen was observed, and it was beaten with a rubber hammer to ensure compaction. The samples, which measured 100 mm × 100 mm × 100 mm, were demolded after 48 h and then cured for 7 d in a box where the temperature and humidity were maintained at 20 °C and 95%, respectively.

### 2.2. Microscopic Parameters

#### 2.2.1. Pore Space Parameter Extraction

**Figure 1:**
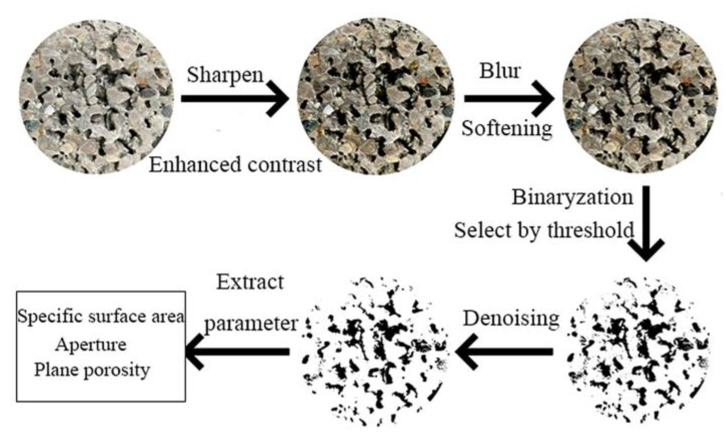
Processing flow of the cross-section maps.

The pervious concrete blocks were cut into eight equal parts using an HQP-150 concrete core sample block cutter (Beijing Zhongbo Ruike Testing Equipment Co., Ltd., Beijing, China). Then, a high-resolution camera was used to capture images of the sections. The captured images were exported to the ImageJ processing software (1.53i). Various operations, such as sharpening, smoothing, and enhancing contrast, were performed to enhance the distinction between the pores and solid parts in the image. The binarization method was adopted, and the grayscale value of the pixels in the image was set to 0 or 255, i.e., the whole image presented a clear black-and-white visual effect to achieve the best view. Consequently, a binary image of the cross-section diagram was obtained, revealing the internal microscopic features of the pervious concrete specimens with different mixing ratios. This process is illustrated in Figure 1.

#### 2.2.2. Matrix Parameter Extraction

**Figure 2:**
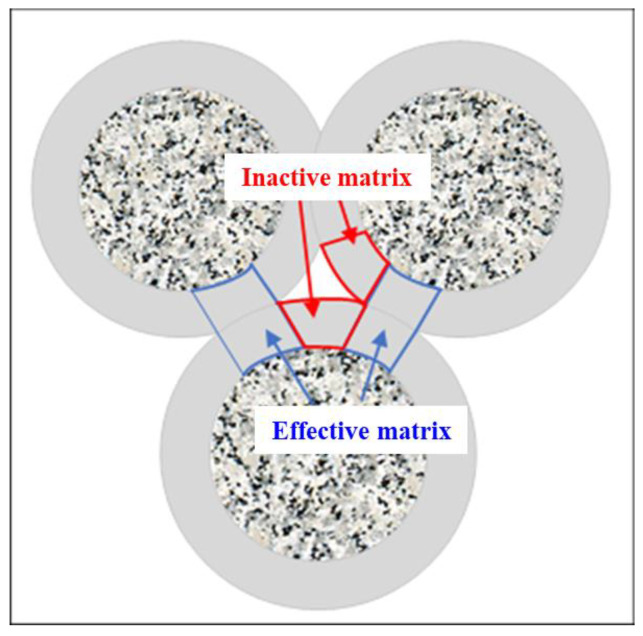
Cement matrix.

Unlike ordinary concrete, pervious concrete has large internal pores, and the strength of a specimen mainly results from the bonding effect of the cement matrix among aggregates. The strength and content of the cement matrix considerably affect the strength of pervious concrete; however, excellent bonding may not be achieved in the entire cement matrix. As shown in Figure 2, the cement matrix uniformly encapsulated the aggregate. When the cement matrix was located between two aggregate particles (i.e., within the blue box in the figure), it bonded the two as a unit. However, the cement matrix shown in the red box had one end in contact with the particle and its other end in the pore space. This part of the matrix also played a role in enhancing strength; however, its effect was considerably less significant than that of the cement matrix located between two aggregate particles. A cement matrix that is created between two particles is called an effective matrix; it can effectively improve the strength of pervious concrete. The cement matrix between the pore space and aggregate particle was called an ineffective matrix; it had an insignificant effect on the strength of the specimen. Moreover, it blocked the internal pores, reducing the pore size; consequently, the pervious concrete’s permeability decreased.

**Figure 3:**
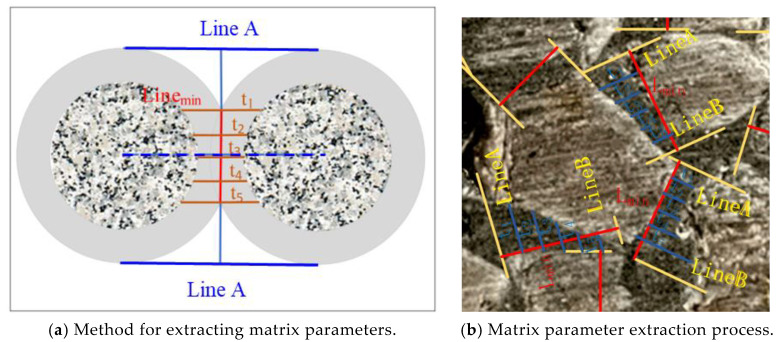
Extraction of the cement matrix parameters.

The pervious concrete used in this study was cut into 10 mm slices and polished on each side before the slices were photographed for calculation. Then, the bonded areas—the cement paste zones that directly connected adjoining aggregates—were manually determined for each section. To assess these bonded regions, line D was initially drawn to indicate the closest distance between neighboring aggregates. Subsequently, two straight lines, *A* and *B*, were drawn parallel to *D*. Lines *A* and *B* were tangent and orthogonal to the contour of the aggregate, respectively. A perpendicular line that intersected with *D* was also drawn and labeled as *L*_min_. The width of the contact zone, denoted as *L*, was measured by considering the portion of line *L* lying in the cement paste; this zone was between lines *A* and *B*. Next, to determine the matrix thickness between two neighboring aggregates, five parallel lines (*t*_1_–*t*_5_) were manually drawn to perpendicularly intersect with line *L*; these lines equally divided line *L*_min_ into six equal intervals. The length of each line within the bonded area was recorded. The average length was used to define the matrix thickness between two adjacent aggregates, as illustrated in Figure 3a. This methodology was employed for each section of the pervious concrete (Figure 3b). The number of matrices, their thickness, and their width were then tallied to obtain the average values.

## 3. Discussion and Analysis

### 3.1. Pore Parameters

#### 3.1.1. Porosity and Pore Size

**Figure 4:**
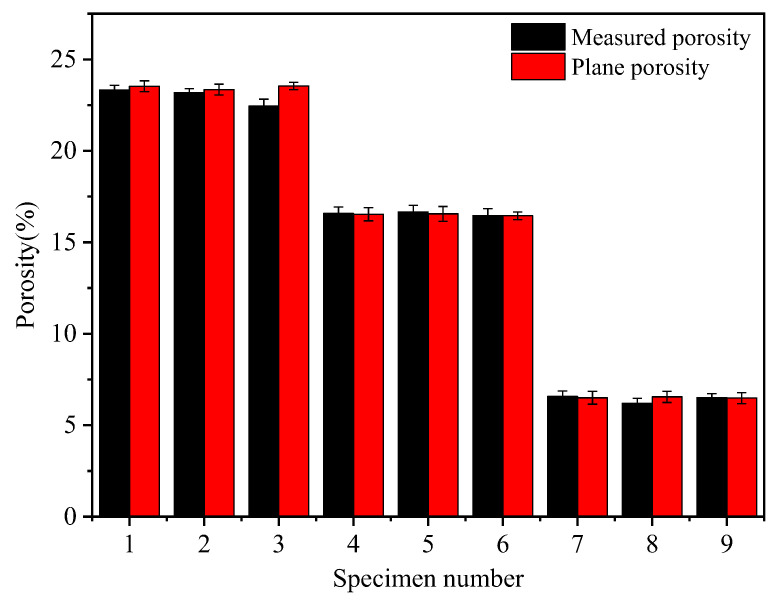
Comparison between the measured porosity and planar porosity.

The measurement of a specimen’s plane porosity involved processing a section of the pore area and calculating the ratio of the pore area to the full area of the section. The average value of the porosity in each section was then obtained to yield the specimen’s plane porosity. As shown in Figure 4, the measured porosity and planar porosity obtained by processing the cross-section diagrams were consistent. This indicated that the microscopic parameters obtained through this approach had a certain level of reliability. Additionally, the porosity of the pervious concrete specimens remained similar when the ash set ratio was the same and the grain size was different. This suggested that the aggregate grain size had a negligible effect on the porosity of the pervious concrete.

**Figure 5:**
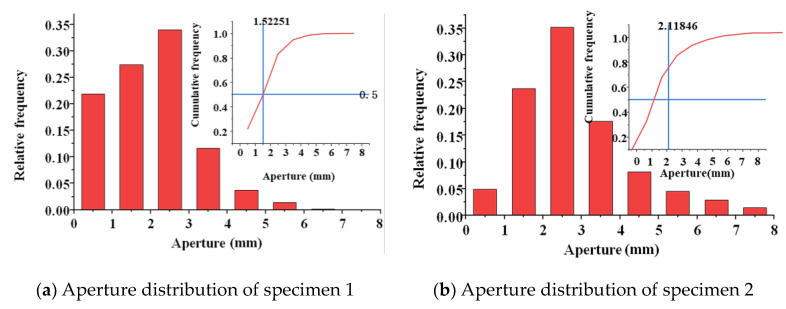

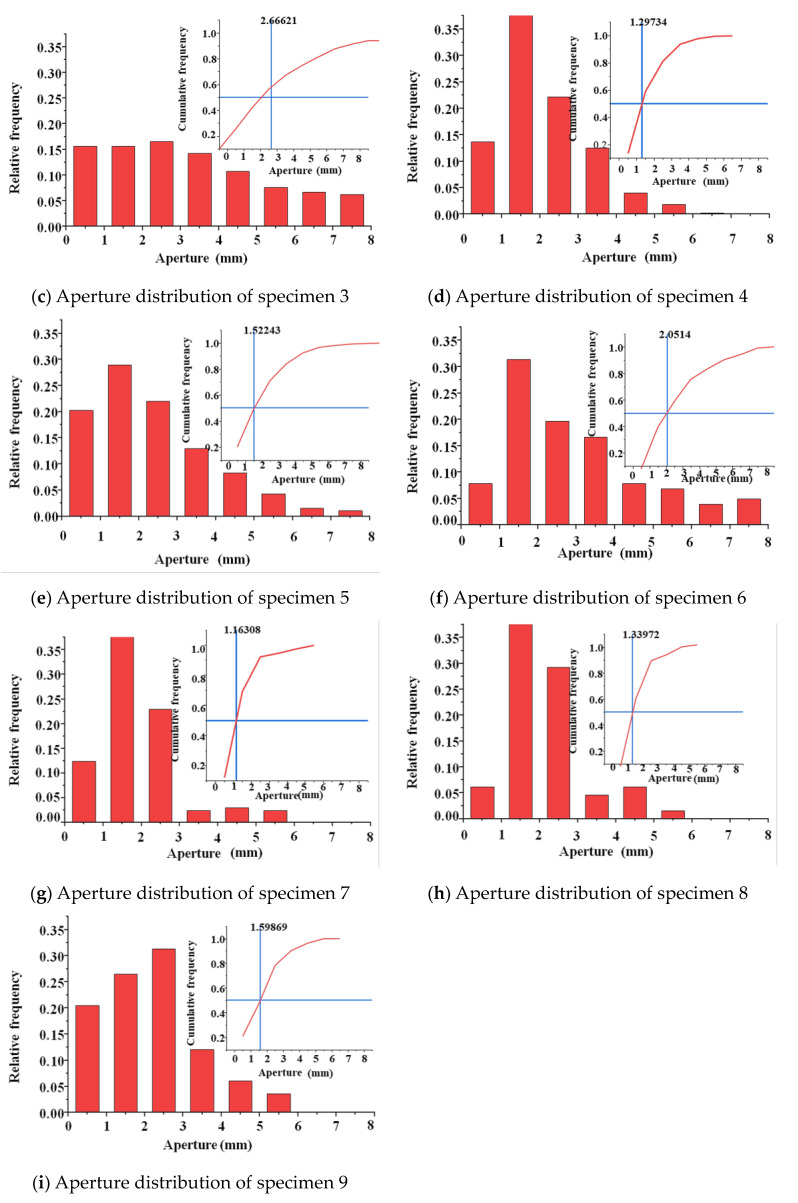
Distribution of aperture frequency.

A frequency histogram of the pore size and particle size distributions of the specimens is shown in Figure 5. The figure indicates that the pore size of the pervious concrete increased with the aggregate size when the ash set ratio remained constant. For specimens with a cement content of 20% and an aggregate particle size of 2.36–4.75 mm, the pore size was concentrated in the range of 1–3 mm. For specimens with an aggregate particle size of 4.75–9.5 mm, the pore size was mainly distributed in the range of 1–4 mm. Furthermore, specimens with aggregate particle sizes in the range of 9.5–16 mm predominantly exhibited pore sizes ranging from 2 to 6 mm. This trend in the pore size distribution was mainly observed in the specimens with cement contents of 30% and 40%. Moreover, increasing the cement content increased the bonding among aggregates, thus dividing the original large and interconnected pores into smaller ones. Consequently, the pore size of the pervious concrete specimens gradually decreased with the increase in the cement content. This was because the increase in the cement content provided enough slurry to cover the surface of the aggregate and fill the voids between the aggregates. To represent the pore sizes of the pervious concrete specimens, the total frequency distribution of the pore size was plotted while considering a pore size with a 50% distribution frequency.

#### 3.1.2. Specific Surface Area

To determine the specific surface area of the porous media to represent the surface area per unit volume, it was necessary to process the images to obtain the pore perimeter of each section from the two-dimensional cross-sections. Then, the two-dimensional images could be stacked along the *z*-axis direction to create a three-dimensional digital model. The perimeters of the pores in each cross-section and the pore area could be considered functions related to the *z*-axis. Let *V*, *δ*, *l*, *S_p_*, and *P_p_* represent the overall volume of a specimen, the internal surface area of the pores, the length along the *z*-axis, the pore area of the cross-section, and the pore perimeter, respectively. With these variables, the specific surface area of the specimen can be expressed using the following equation:

(1) $$S=\delta /V=[\int_{0}^{l}P_{p}(y)dy]/V$$

The integral part of Equation (1) can be approximately solved using the mechanical product method. This method uses discrete two-dimensional image data, as expressed in Equation (2):

(2) $$\int_{0}^{l}f(y)dy\approx \sum_{k=0}^{n}A_{k}f\left(y_{k}\right)$$

The location of the product node is represented by *y_k_*, whereas *A_k_* indicates the weight of this node.

This equation indicates that the pore perimeter of a pervious concrete specimen can be measured using several cross-sectional drawings. The overall specific surface area can then be calculated using the numerical integration method. Several commonly used mechanical integration formulas are available for this purpose. They include the composite trapezoidal formula (Equation (3)), composite Simpson formula (Equation (4)), and composite Cotes formula (Equation (5)):

(3) $$T_{n}=\left[f(a)+2\sum_{k=1}^{n-1}f\left(x_{k}\right)+f(b)\right]h/2$$

(4) $$S_{n}=\left[f(a)+4\sum_{k=0}^{n-1}f\left(x_{k+1/2}\right)+2\sum_{k=1}^{n-1}f\left(x_{k}\right)+f(b)\right]h/6$$

(5) $$C_{n}=\left[7f(a)+32\sum_{k=0}^{n-1}f\left(x_{k+1/4}\right)+12\sum_{k=0}^{n-1}f\left(x_{k+1/2}\right)+32\sum_{k=0}^{n-1}f\left(x_{k+3/4}\right)+14\sum_{k=1}^{n-1}f\left(x_{k}\right)+7f(b)\right]h/90$$

**Figure 6:**
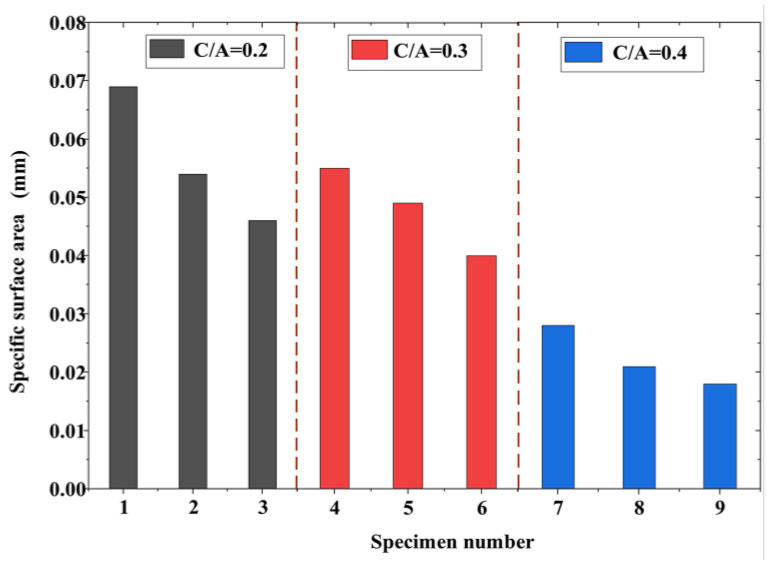
Specific surface area.

When the ash set ratio differed, the specific surface area of the pervious concrete exhibited noticeable variations based on the porosity, as illustrated in Figure 6. Similarly, the specific surface area significantly differed when the particle size varied with the same ash set ratio. This observation indicated that both the pore size and porosity had a substantial impact on the specific surface area. First, a large porosity corresponded to more pores, resulting in a high total perimeter of pores in the cross-section and a higher specific surface area per unit volume. Moreover, for specimens with the same porosity, large pores were present in aggregate blocks with large sizes. In contrast, reducing the particle size of the aggregate caused large pores to fragment into multiple smaller pores. Consequently, the specimens with small aggregate sizes exhibited a larger specific surface area with the same planar porosity.

### 3.2. Substrate Parameters

#### 3.2.1. Number of Effective Matrices

**Figure 7:**
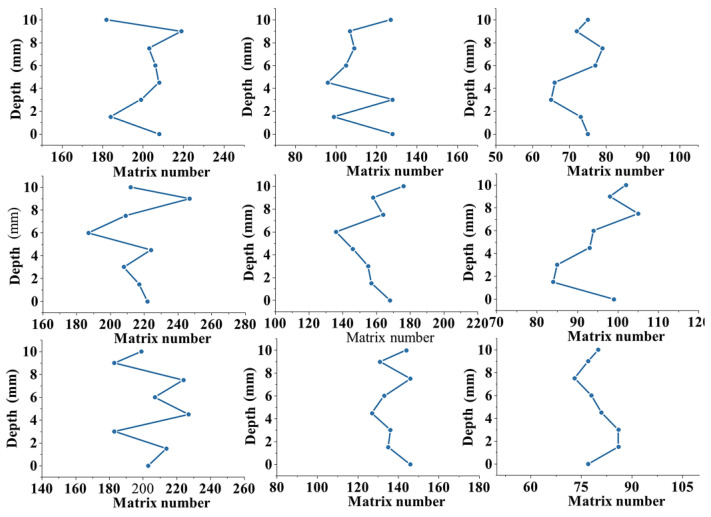
Variations in substrate quantity.

The change in the number of cement matrices within the pervious concrete was observed by quantifying the matrices in each section and plotting the change along the depth of the specimen (Figure 7). The increase in the cement content affected the number of bonded matrices; however, the magnitude of the change remained constant. An exception to this was when the aggregate particle size was 2.36–4.74 mm, and the cement content increased from 20% to 30%, significantly increasing the bulk of the bonded region. This can be explained by the fact that specimens with small particle sizes and high porosity have large specific surface areas, and they require a sufficient number of cement matrices for the complete encapsulation of the aggregate. When the cement content was 20%, the cement did not fully encapsulate the aggregate. The small pores connected, forming larger pores and reducing the number of encapsulating cement matrices. However, the increase in the cement content from 20% to 30% caused the cement to split the large pores, forming independent small pores and increasing the number of bonded matrices. In contrast, when the aggregate particles were large, the number of bonded matrices significantly decreased. For particle sizes in the ranges of 2.36–4.75, 4.75–9.5, and 9.5–16 mm, the numbers of matrices were in the ranges of 210–220, 100–160, and approximately 80, respectively. In conclusion, the characteristics and thickness of the cement paste coating had negligible effects on the number of bonded matrices. In the pervious concrete, the number of such matrices was mainly influenced by the aggregate size; the nature of the cement paste did not play a significant role.

#### 3.2.2. Width of the Effective Matrix

**Figure 8:**
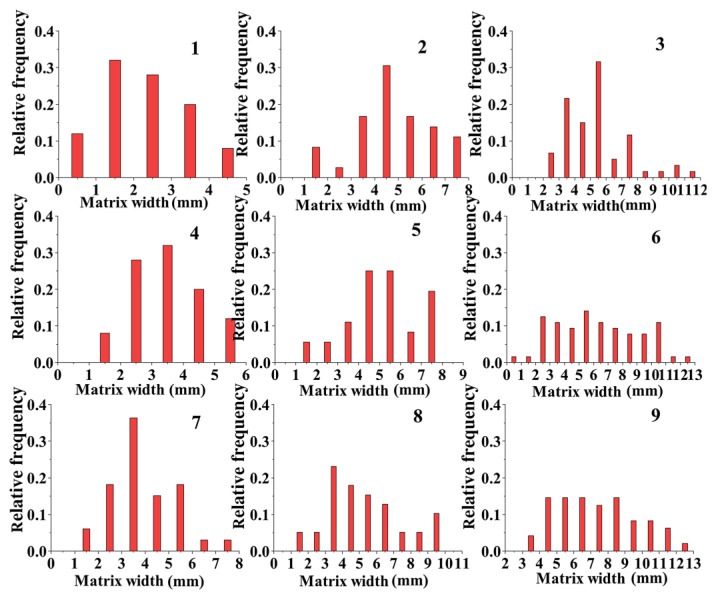
Matrix width.

This test was conducted on each section of the pervious concrete specimens to obtain statistics on the width of the matrices among the aggregate particles (Figure 8) and to remove the influence of ineffective matrices. As shown in Figure 8, the cement content significantly affected the matrix width. As the cement content increased, the number of cement matrices that were generated increased, which increased the thickness of the bonding matrices around aggregate particles and the effective bond length between two adjacent aggregate particles. Additionally, the particle size of the aggregates considerably affected the matrix width of the pervious concrete specimens. Small aggregates, which had a large specific surface area, required more cement for complete encapsulation than large aggregates did. However, when the cement content reached a certain level, this effect gradually diminished. Consequently, the matrix width of the specimens in groups 7 and 8 exhibited a relatively large increase. Moreover, the specimens with large aggregate particles had a wide distribution of matrix widths. The matrix width of the specimens in groups 3, 6, and 9 ranged from 1 to 13 mm, whereas that of the specimens in groups 2, 5, and 8 ranged from 1 to 10 mm. In contrast, the matrix width of the specimens in groups 1, 4, and 7 only ranged from 1 to 8 mm. Group 7 had a matrix width exceeding 5 mm, and the maximum matrix width in the remaining two groups was less than 5 mm.

#### 3.2.3. Effective Matrix Thickness

The matrix thickness was perpendicular to the width and length of the bonding matrix. However, the bonding matrix was not uniformly distributed along the matrix width. Consequently, the direct determination of the matrix thickness was impossible. To overcome this problem, the bonding band needed to be partitioned. As shown in Figure 3, the bonding matrix was evenly divided into six equal parts in a direction perpendicular to the matrix width (excluding lines *A* and *B*). The representative value of the bonding matrix was calculated by obtaining the average value of t from the other five lines; t was calculated using Equation (6).

(6) $$t=(t_{1}+t_{2}+t_{3}+t_{4}+t_{5})/5$$

**Figure 9:**
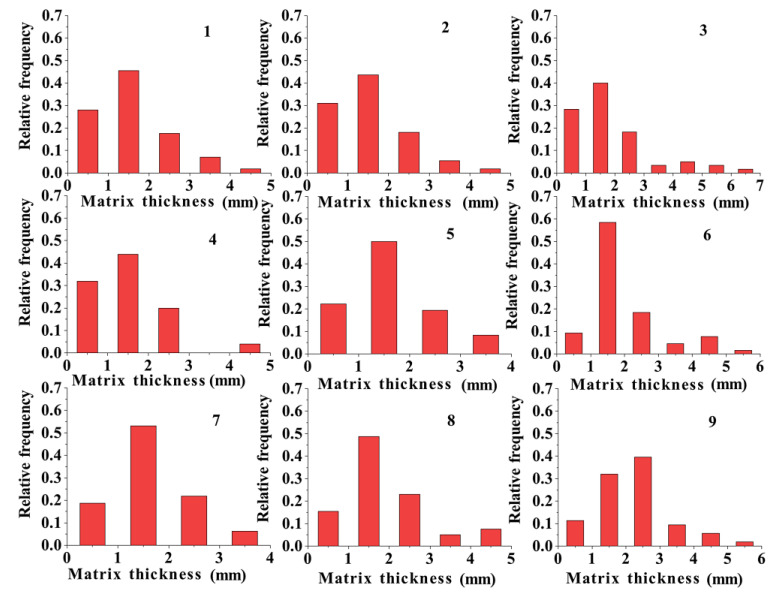
Distribution of the effective substrate thickness.

Compared with the range of the matrix width distribution, that of the matrix thickness was generally smaller, as shown in Figure 9. When the aggregate particle size was the same, the highest values of the thickness distribution were all in the range of 1–2 mm (except for that of group 9). However, as the particle size increased, the range of the thickness distribution gradually increased. Therefore, the effect of the aggregate particle size on the thickness of the bonded matrix was greater than that of the cement content. The matrix thickness of the specimens in all groups (except for those with a particle size ranging from 9.5 to 16 mm) was less than 2. In groups 3, 6, and 9, when the particle size was 9.5–16 mm, the matrix thickness values were 2.46, 2.82, and 3.12 mm, respectively.

### 3.3. Coefficient of Permeability

#### 3.3.1. Effect of the Pore Size on the Permeability Coefficient

**Figure 10:**
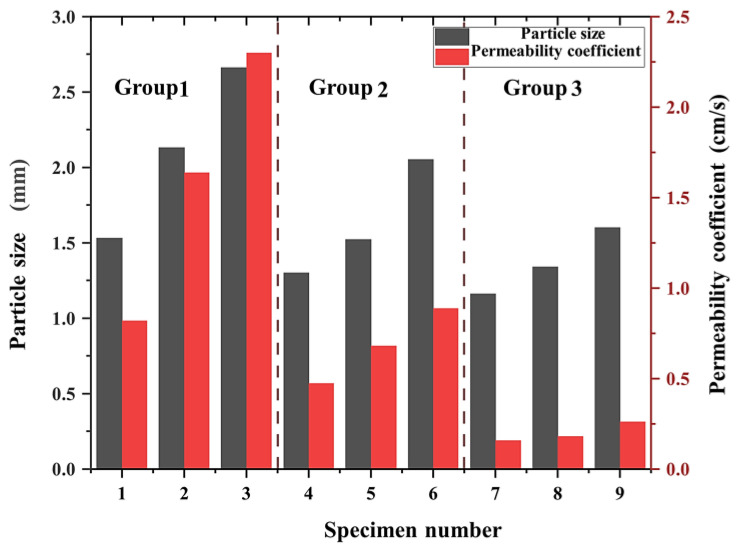
Relationship between the permeability coefficient and pore size.

The permeability coefficient of the pervious concrete specimens varied even when the same group of specimens had the same porosity. The relationship between the permeability coefficient and pore size is depicted in Figure 10.

As shown in Figure 10, the permeability coefficient of the pervious concrete increased with the pore diameter. For instance, in group 1, when the pore diameter was 1.53 mm, the permeability coefficient was 0.82 cm/s. When the pore diameter increased to 2.13 and 2.66 mm, the permeability coefficients increased to 1.64 and 1.72 cm/s, respectively. Similarly, in groups 2 and 3, the permeability coefficient of the pervious concrete increased with the pore diameters. This can be explained by the fact that, although the porosity of the test block remained the same, a larger pore diameter provided a wider internal percolation channel. Lu et al. [27] found that, with the same porosity, a larger aggregate size or pore size could increase the pore connectivity factor, and Lima et al. [28] also argued that permeable concrete had greater permeability due to its larger pore size and greater connectivity, which was in agreement with the findings in this study. As a result, the percolation path became less zigzagged, and the time taken for the passage of water through the channel was reduced. Consequently, more water could flow through the channel per unit of time, resulting in a larger permeability coefficient. In contrast, a small pore diameter created a narrow internal percolation path, limiting water flow. Water tension prevented the flow of water through the seepage channels of pores of a small size, rendering the path even more tortuous. Thus, a small pore size led to a narrow internal seepage path and increased the water tension, resulting in a tortuous path that hindered water flow.

#### 3.3.2. Effect of the Porosity and Specific Surface Area on the Permeability Coefficient

Currently, the most commonly used permeability model for porous media is the Kozeny–Carman (KC) equation (Equation (7)). For the accurate assessment of the permeability properties of the pervious concrete and the influences of the parameters on these properties, the following functional relationship between the microscopic parameters and the permeability coefficient needed to be established:

(7) $$k=\varphi^{3}/S^{2}$$

where *k* is the permeability coefficient of the porous media; *φ* is the porosity; *S* is the specific surface area.

The foregoing permeability equation is the KC equation with fractal characteristics. It indicates that permeability is influenced by the fractal dimension of the pore structure of the porous medium, as well as the macroscopic physical parameters and microscopic pore structure parameters. In contrast to the conventional KC equation, this equation incorporates parameters that are related to the fractal dimension of the porous medium and the microscopic pore structure parameters. To establish Equation (8), a new parameter, *α*, was introduced (*α* is defined as the ratio of the third power of porosity to the square of the specific surface area):

(8) k=1/α

**Figure 11:**
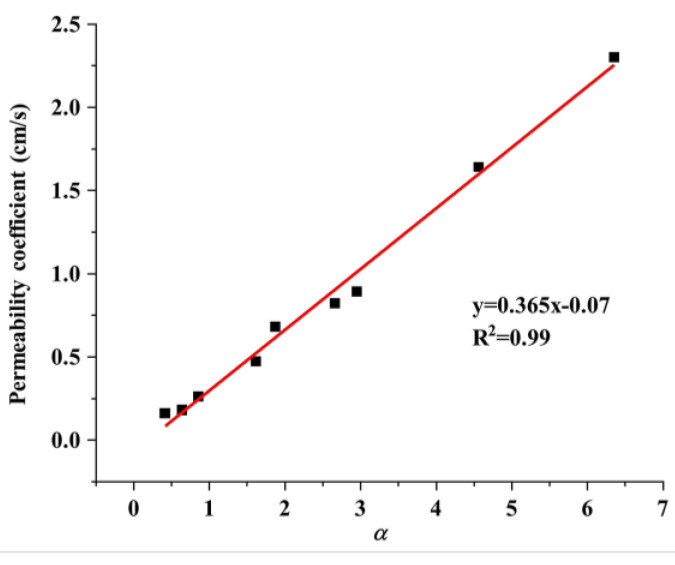
Relationship between the permeability coefficient and pore parameters.

The relationship between the permeability coefficient and the pore parameter is depicted in Figure 11. The figure illustrates that the two variables had a positive correlation. Specifically, the permeability coefficient could be calculated as the ratio of the third power of porosity to the square of the specific surface area. However, note that the permeability coefficient derived from the macroscopic tests increased with the porosity and decreased with the specific surface area (assuming that the porosity remained constant).

The larger the pore diameter, the smaller the specific surface area of the specimen, as shown by the values listed in Table 5. This indicated that when the porosity was the same, an inverse relationship between the pore diameter and specific surface area existed [29,30,31]. Furthermore, the specific surface area could be inferred to decrease as the permeability coefficient increased. This trend aligned with established research findings, thus confirming that the permeability coefficient was positively correlated with the pore diameter.

### 3.4. Compressive Strength

#### 3.4.1. Matrix Area

To effectively assess the strength of the pervious concrete, the establishment of the relationship between the pervious concrete and its bonding matrix was required. Previous studies [32,33,34,35] focused on the relationship between strength and pore parameters, such as the porosity and pore diameter. However, the bonding matrix is the main source of strength in pervious concrete. The parameters, quantity, width, and thickness of the bonding matrices were extracted in previous sections to obtain relevant information. However, the width, thickness, and area of a single matrix only indicate the strength of the bond between two aggregates. To accurately assess the strength of an entire pervious concrete section, it was necessary to calculate the effective bonding matrix throughout the section. To accomplish this, the total width and area of the matrix in each specimen section were determined by combining the average width and thickness of the matrix in each specimen with the number of matrices present.

In each section, the representative width of the substrate was obtained by averaging the substrate width. The total width of the substrate in each section was calculated as the product of the average width of the substrate and the number of substrates in that section. Equations (9) and (10) were used to determine the total width of the substrate in each section and the representative width of the substrate in the specimen. Furthermore, the total area of the effective matrix of the pervious concrete was obtained by multiplying the total width and average thickness of the matrix:

(9) $$L_{t}=N\times L$$

(10) $$A_{t}=t\times L_{t}=t\times N\times L$$

where *N*, *L*, *L_t_*, *t*, and *A_t_* denote the number of substrates, the width of a substrate, the total width of all substrates in a particular section, the thickness of a substrate, and the total area of all substrates in the section, respectively.

**Figure 12:**
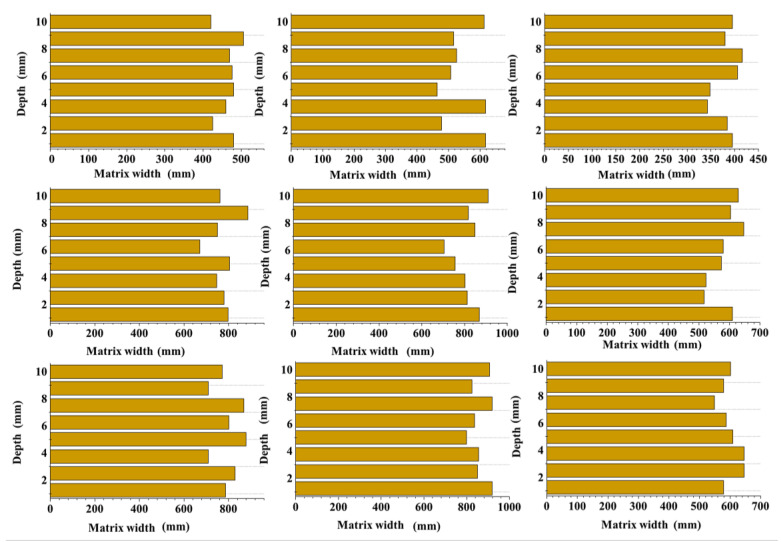
Matrix width distribution.

As shown in Figure 12, the cement matrix width along the section was uniformly distributed without large fluctuation amplitudes. This indicated that the pervious concrete was adequately inserted and compacted during the preparation process. Furthermore, the cement matrix was uniformly distributed along the height, and the strength of each section was relatively consistent. Therefore, by calculating the average width and area of each section, equations for accurately representing the overall condition of the specimen could be derived.

**Figure 13:**
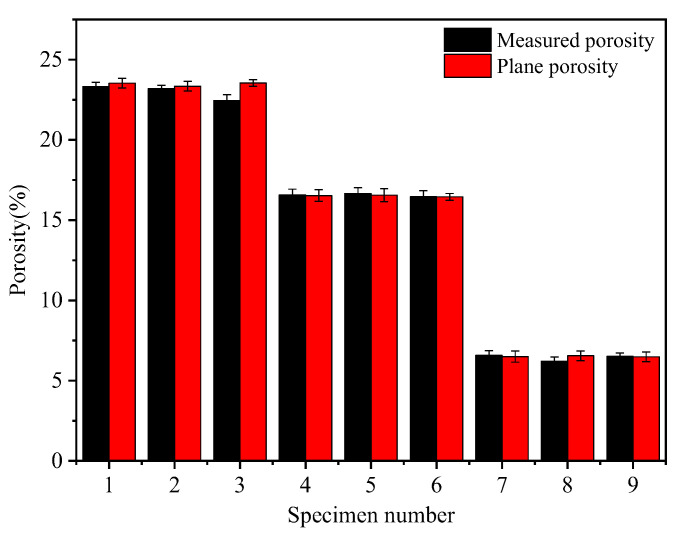
Total width and area of matrices.

As shown in Figure 13, for the same ash–aggregate ratios, the pervious concrete specimens had the largest total matrix width and total area when the particle size range was 4.75–9.5 mm. In contrast, the specimens with a particle size ranging from 9.5 to 16 mm have the smallest matrix area despite having the largest average matrix width and thickness. This was because large particle sizes resulted in a small number of aggregates in the unit area. Although large particles were favorable for improving the attachment of the cement matrix, small particles had more aggregates per unit area, increasing the matrix quantity and contact zones among aggregates (Figure 7). Specimens with small aggregates had considerably more matrices specimens with large aggregates did. Furthermore, when the ash–aggregate ratio increased from 0.2 to 0.3, both the effective area and width of the cement matrix significantly increased. However, when the ash–aggregate ratio increased from 0.3 to 0.4, the rates of increase in the effective matrix area and matrix width decelerated despite the same increase occurring in the cement content. This indicated that most of the increase in cement matrices in the latter group was due to ineffective cement matrices. They did not significantly improve the strength of the specimen; instead, they blocked the pore space, resulting in a significant decrease in the permeability coefficient. These findings were consistent with the results observed in the cross-section structure, supporting the validity of assessing the macroscopic properties of pervious concrete based on microscopic parameters.

#### 3.4.2. Effect of Matrix Parameters on Compressive Strength

Equation (11), which relates the pervious concrete specimens and the matrix width, was derived by fitting the test data.

(11) y=2.8×exp(0.021x)−1

**Figure 14:**
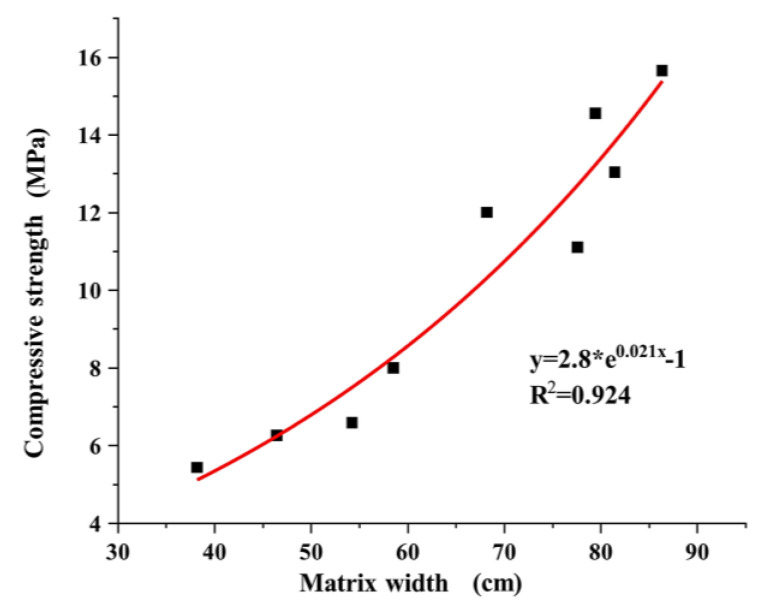
Relationship between the compressive strength and matrix width.

The compressive strength of the pervious concrete was observed to increase with the effective width of the matrix, as depicted in Figure 14. A gradual increase in the slope of the curve could be observed. This effect could be attributed to the enhanced strength of the pervious concrete resulting from an increase in the effective matrix content. However, note that the total number of matrices, which played a crucial role in the strength of the specimens, was determined by the particle size. When a bonding matrix occupied the part of the aggregate requiring bonding, the pervious concrete attained sufficient strength while maintaining a certain permeability coefficient. Nonetheless, a further increase in cement content could cause excess cement to fill the pore space. Although this could improve the strength of pervious concrete to some extent, the permeability of the concrete could decrease. The test data supported this observation, demonstrating that the increase in strength was significantly less when the cement content was increased from 0.3 to 0.4. This differed from the increase in strength observed when the cement content was increased from 0.2 to 0.3. Therefore, in practical engineering applications, a high matrix content is advisable within the range of the total effective matrix to ensure a sufficient strength for pervious concrete.

**Figure 15:**
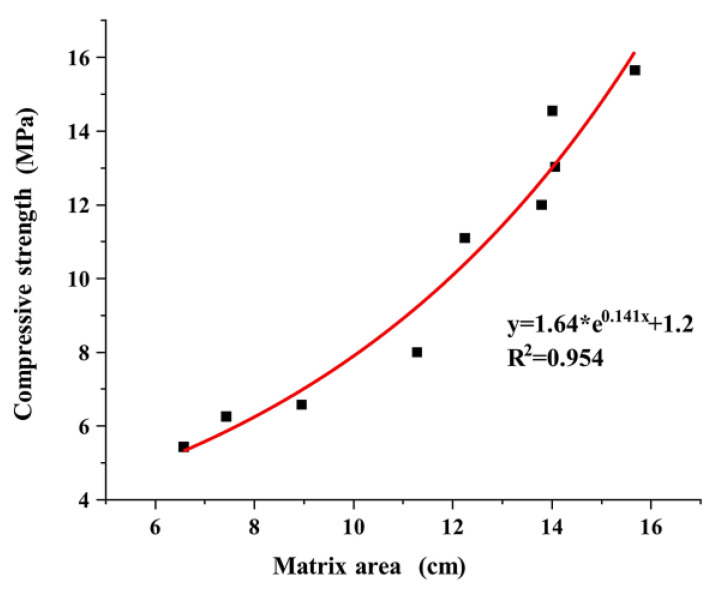
Relationship between the compressive strength and matrix area.

In Figure 15, the plotted curves, which have the same trend as that of the curves in Figure 14, show the relationship between the compressive strength of the pervious concrete specimens and the total effective matrix area. This indicated that no significant differences in the effective matrix thickness occurred inside the pervious concrete specimens across the different cases; this observation is also reflected in Figure 9. Therefore, when the matrix strength was determined, the use of large aggregate particles did not effectively increase the thickness of the matrix inside the concrete specimens. On the contrary, they reduced the concrete strength by decreasing the number of matrices and the total area.

The strength of pervious concrete emanates from its bond matrices, which are influenced by the aggregate content and properties of the matrices. Consequently, the strength of the specimens increased with the number of matrices. Specimens with large aggregate sizes had a less significant increase in matrix thickness, which decreased the matrix strength due to the reduced bonding area. These findings were consistent with those obtained in the macro-tests. A small aggregate particle size also resulted in reduced strength because the increased surface area of the aggregates required more matrix encapsulation, resulting in narrow and short widths among the aggregates. Although previous studies demonstrated that a decrease in strength occurred with the use of large particle sizes, only a few investigated aggregates that were approximately 2 mm in size. Therefore, reducing the aggregate particle size can increase the strength of pervious concrete; however, excessively small particles can have the opposite effect.

## 4. Conclusions

The relationships among the aggregate particle size, mixing ratio, porosity, and microscopic parameters in the process of preparing pervious concrete were investigated in this study. Pervious concrete was made from three groups of single primary mixtures of aggregates to analyze the factors influencing its performance from a microscopic perspective. The main conclusions were as follows.

1. The feasibility of the research method was verified by the high consistency between the planar porosity obtained from the cross-section diagrams and porosity measured in tests. The test block’s internal pore diameter increased with the aggregate particle size; however, it decreased with the ash set ratio. Furthermore, a high porosity value corresponded to a large specific surface area. Conversely, when the porosity was the same, a large pore diameter resulted in a small specific surface area.
2. When the ash set ratio was increased, the large pores were divided into smaller pores, increasing the number of cement matrices. Additionally, the smaller the aggregate particle size, the greater the number of matrices within the pervious concrete specimen. The effective matrix width was influenced by both the cement content and the aggregate particle size, and an increase in both factors resulted in a wider matrix. The matrix thickness was affected by the particle size; large particles resulted in a thicker matrix. Notably, the variations in cement content had minimal influence on the thickness of the cement matrix.
3. The permeability coefficient of the pervious concrete was directly proportional to the product of the third power of porosity and the square of the specific surface area. Additionally, the permeability coefficient increased with the pore diameter. In contrast, the compressive strength of the pervious concrete was exponential relative to the matrix parameters. Specifically, the larger the total thickness and total area of the effective matrix, the greater the pervious concrete’s compressive strength.

## Figures and Tables

**Table 1:** Chemical composition of the cement.

Chemical Composition	SiO_2_	SiO_3_	Fe_2_O_3_	Al_2_O_3_	CaO	MgO	TiO_2_	LOI
Content/%	21.45	2.75	2.40	5.15	63.40	1.5	0.19	1.50

**Table 2:** Parameters of the coarse aggregate.

Particle Size Range/(mm)	Sludge Content/%	Apparent Density/(kg/m^3^)	Compact Bulk Density/(kg/m^3^)	Porosity/%
2.36–4.7	0.5	2802	1471	48.8
4.75–9.5	0.5	2788	1452	47.3
9.5–16	0.5	2709	1429	45.6

**Table 3:** Parameters of the polypropylene fiber.

Density/(g/cm^3^)	Diameter/(μm)	Length/(μm)	E/MPa	Elongation/%	Tensile Strength/MPa	Water Absorption/%
0.94	3~4.5	120	4.2 × 10^3^	9.3	3950	<0.1

**Table 4:** Design of the specimen mix ratio.

No.	Aggregate Particle Size	Water–Cement Ratio	Ash Set Ratio	Fine Aggregate
1	2.36–4.75 mm	0.36	0.2	10%
2	4.75–9.5 mm	0.36	0.2	10%
3	9.5–16 mm	0.36	0.2	10%
4	2.36–4.75 mm	0.36	0.3	10%
5	4.75–9.5 mm	0.36	0.3	10%
6	9.5–16 mm	0.36	0.3	10%
7	2.36–4.75 mm	0.36	0.4	10%
8	4.75–9.5 mm	0.36	0.4	10%
9	9.5–16 mm	0.36	0.4	10%

**Table 5:** Results of microscopic tests.

No.	Measured Porosity (%)	Calculated Porosity (%)	Average Pore Size (mm)	Specific Surface Area (mm^2^/mm^3^)	Width of Matrix (mm)	Matrix Quantity	Matrix Thickness (mm)	Permeability Coefficient (cm/s)	Compressive Strength (MPa)
1	23.62	23.31	1.53	0.069	2.29	201	1.60	0.82	6.26
2	23.47	23.70	2.13	0.054	4.79	112	1.65	1.64	6.58
3	22.94	23.79	2.66	0.046	5.27	73	1.72	2.30	5.43
4	16.50	16.98	1.30	0.055	3.59	216	1.57	0.47	11.1
5	16.71	16.52	1.52	0.049	5.13	158	1.71	0.68	13.03
6	16.29	16.78	2.05	0.04	6.16	95	1.92	0.89	8
7	6.51	6.88	1.16	0.028	3.87	205	1.68	0.16	14.55
8	6.49	6.56	1.34	0.021	5.29	137	1.91	0.18	15.65
9	6.58	6.52	1.60	0.018	7.50	80	2.12	0.26	12

## Data Availability

Data are contained within the article.
